# Managing emotionally charged conversations between faculty and residents: a skill-building workshop

**DOI:** 10.15694/mep.2018.0000080.1

**Published:** 2018-04-10

**Authors:** Agatha Parks-Savage, Amelia Wallace, Cynthia P Cadieux, Linda R Archer

**Affiliations:** 1 Eastern Virginia Medical School

**Keywords:** Challenging conversations, Remediation, Feedback, Workshop development

## Abstract

This article was migrated. The article was marked as recommended.

**Background:** Discussions with residents regarding remediation, probation, or dismissal are challenging, but only limited practical guidance about constructive conversations is available.

**Aims:** To provide useful assistance to those who must conduct such conversations.

**Methods:** Literature review, surveys, workshop development.

**Results:** The workshop offers guidance about conducting challenging conversations, including opportunities for role playing.

**Conclusions:** Development of the workshop is ongoing, and initial feedback recognizes its importance and applicability to help faculty manage challenging or confrontational conversations.

## Introduction

Confrontation is conversationally complex and involves the limbic system, cognitive strategies, and facilitative skills that transcend normal conversational habits ( Krauss and Morsella 2014; Lindner 2014; Deutsch 2014). Discussions with residents regarding remediation, probation, or dismissal may escalate to confrontations, and faculty charged with managing these conversations may mitigate the message to the extent that the resident is not fully aware of the problem ( Jackson et al. 2015). Further, the resident’s conversational style may not parallel that of the program director, and personality differences can be sources of conflict ( Tannen 2005; Sandy et al. 2014). Conversations regarding remediation, probation, or dismissal involve high stakes and thus potentiate fear, anger, humiliation, and guilt, which can contribute to conflict ( Lindner 2014). Often, residency/fellowship program directors conduct these conversations based on compiled data that include perspectives of others and themselves. Sharing multiple perspectives can be challenging because some of the contributing perspectives may be subjective ( Bhatti et al. 2016). Additionally, these conversations must comply with guidelines of the Accreditation Counsel for Graduate Medical Education (ACGME) (see http://www.acgme.org/About-Us/Policies-and-Related-Materials). When formalized by documentation, conversations can become highly sensitive for most residents and create conversational resistance ( Guerrasio et al. 2016).

Published sources widely address remediation of ACGME core competencies, but few outline the process of having conversations with residents regarding remediation, probation, and dismissal. Guidance regarding questions that program directors should prepare is available, but without specifics regarding techniques to facilitate difficult conversations ( Liu et al. 2016). Current literature regarding resident remediation supports the need for additional exploration regarding conducting challenging conversations with residents, and professionalism is the ACGME core competency most associated with difficulties regarding remediation ( Paglia and Frishman 2011). Perhaps because core competencies like medical knowledge have abundant support tools, remediation in this area is more successful ( Paglia and Frishman 2011). On the topic of communication in general and conflict mitigation in particular, studies often focus on the behavioral attributes of a conversation and emphasize the need for self-awareness during communication ( Patterson et al. 2002). In these contexts, emotional engagement and involvement in conversational outcomes are necessary to explore the stakes of highly charged conversations.

At Eastern Virginia Medical School (EVMS), the Office of Graduate Medical Education (GME) identified challenges regarding managing conversations involving remediation, probation, and termination. The Designated Institutional Official (DIO) and Assistant Dean developed a quality improvement workshop to help faculty navigate these challenges. The first workshop took place at the ACGME Annual Educational Conference in Orlando, FL, in March 2017.

## Workshop development

Because of its experiential, flexible nature, role-playing was the instructional method selected for the workshop ( Coleman and Prywes 2014). In addition, we used standardized patient (SP) simulation as a training method for developing communication skills in a variety of contexts ( Epstein 2007) and providing experiential role-playing ( Copleman and Prywes 2014). Additional value in using SPs arises because SPs can provide feedback as “residents” in the conversation ( Dayer Berenson et al. 2012).

We used 2 surveys in developing this project. Program directors and associates were asked about challenging conversations, conversational preferences, and stressors experienced during conversations with residents. Items included questions about the confrontational preference of the participant ( Raines 2013), resident personas or affects that were challenging, and the frequency of conversations about remediation, probation, and termination.

The ACGME sent out postsurveys on general conference satisfaction and relevance of the workshop. An additional 5-question postencounter survey of workshop participants about crucial conversations and the use of simulation in future workshops was sent by the workshop presenters. These items were formulated using the survey question guide from the Association for Medical Education in Europe ( Artino Jr et al. 2014). Additional survey questions were added to further explore conversational style and the use of simulation.

Three video recordings were developed for use in the workshop. Each was 3-4 minutes long and presented vignettes of crucial conversations between role-playing faculty and residents. The affect of the simulated residents in the recordings were 3 personality types identified by the DIO, Associate Dean, and EVMS survey respondents as the most challenging personality types: passive-aggressive, sad/depressed, and aggressive. The contexts of the scenarios in the videos were remediation, probation, and termination.

Medical knowledge and professionalism are the most prevalent causes for remediation and probation ( Guerrasio et al. 2016; Liu et al. 2016). Because of the communication challenges inherent in working with someone who scores low in professionalism ( Liu et al. 2016; Hoffman et al. 2016), the latter was the core competency most used in the simulation design. The cases were developed in collaboration with the Assistant Dean of GME, and the contexts and personalities of the simulated residents were similar to those we see with other residents across medical disciplines. All 3 resident portrayals were enacted by postgraduate year 2 residents who demonstrated behaviors that indicated issues with professionalism. Details of the simulation varied slightly to include different challenges typical of a resident’s demeanor. The 3 cases were: a passive-aggressive resident called to the office for a conversation about remediation, a depressed/crying resident being put on probation, and a verbally/physically aggressive resident being dismissed from the program.

The passive-aggressive resident simulation included conversational challenges intended to engage the learner’s limbic system: the resident’s answering his cell phone during the conversation, incongruent verbal and nonverbal behaviors (eg, indicating understanding and respect but nonverbally conveying disinterest), and lack of insight regarding the challenges. Workshop attendees were oriented to his demeanor by being told this resident historically was not liked by peers, was found to be a weak colleague, and had been recently fired by a patient’s family for being overheard making rude comments about their concerns.

The conversational challenges of the depressed/crying resident included negotiating not to be put on probation, crying, disclosure of personal information to elicit sympathy (eg, “I’m the first person in my family who went to college” or “My fiancé recently ended our engagement”). The orienting information for workshop participants described this resident as highly likeable and high-scoring regarding medical knowledge but recently making mistakes regarding follow-through with patients and errors ordering tests.

The simulation regarding the verbally/physically aggressive resident was inherently challenging because the objective was to dismiss the resident from the program. Conversational challenges included lack of insight and refusal to accept the decision, interrupting the program director, threat of litigation, and physical indicators of aggression (eg, standing, leaning forward, and sharp hand gestures). For this simulation, the learners worked in pairs in which one was the DIO and one was the program director addressing the concerns with the simulated resident. To focus the conversation on the dismissal, pairs were instructed that all paperwork leading to the dismissal of this resident was complete and approved by the appropriate institutional parties.

A prevalent theme in conflict resolution theory is developing a reciprocal conversation and ensuring the perspectives of all parties are explored and integrated ( Patterson et al. 2002). Reciprocal conversations and conversations that promote reflection are also strategies to aid in developing professionalism and interprofessional communication ( Patterson et al. 2002). In the context of the 3 scenarios just outlined, the conceptual framework for the workshop was based on conflict resolution theory, interpersonal psychology, and enhanced communication techniques, including the 7 steps outlined by Patterson et al for managing crucial conversations ( Patterson et al. 2002) and 2 mnemonic devices from the Master Interview Rating Scale (unpublished data, EVMS). The 7 steps blend perspective-sharing with techniques to build dialogue to move the conversation forward ( Patterson et al. 2002). During the workshop we encouraged practice using 2 communication devices, specifically the mnemonic devices of FIFE (feelings, ideas, function, and expectations) and NURS (name emotion, understanding, respect, and support). These 2 techniques provided steps for eliciting perspectives and verbalizing empathy by building on the steps supplied by Patterson et al (2002).

The workshop design centered on SP simulation using standardized residents instead of standardized patients. The workshop was designed for 90 minutes (45 minutes of didactic presentation, including videos, and 45 minutes of live simulation) and, as noted, was piloted at the annual ACGME conference for an audience of approximately 200 participants (including DIOs, program directors, and faculty from various US institutions). The simulation participants were 6 volunteers from the audience, 2 each for discussions regarding remediation, probation, and dismissal. To promote audience/observer engagement, 1 presenter circulated the room with a handheld microphone to facilitate conversations.

During simulations, volunteers were asked to interact with the standardized residents (the trained PGY2 residents from EVMS) and then to come out of role and share the challenges they experienced during the simulated conversations. Volunteers who worked with the passive/crying resident reported challenges engaging in the simulation because they wanted more background data (this may be an inherent limitation in simulations). The audience noted that many volunteers used the 7 steps of FIFE and NURS (reviewed during the earlier didactic portion of the workshop). Upon reflection, volunteers noticed that they did, in fact use these techniques but did so reflexively.

Volunteers who worked with the passive-aggressive resident demonstrated techniques that were useful in redirecting his behavior. During debriefing, the volunteers reported feeling stressed and annoyed with the resident while talking with him about amending his behaviors.

The last simulation involved the resident who was being terminated. For this simulation, 2 volunteers were recruited, one of whom enacted the DIO and one of whom played the program director. They were instructed to work as a team to talk with the resident. During the simulation and as the resident became agitated, one of the volunteers demonstrated physical indications of stress (rising volume and pitch of voice, arms crossed tightly across chest, and directive statements and hand gestures in response to the standardized resident’s affect). The facilitator paused the simulation and oriented participants toward the safety of the simulation, highlighting that volunteers were not in physical danger. In simulation, this is significant because the response of the volunteers and observers indicated that the simulation was providing the needed stimulation for participants to practice navigating conversations while emotionally engaged.

Another characteristic of this workshop was the facilitated discussion involving presenters, standardized residents, volunteers, and observers. The facilitated conversations led to reflection and insight building among the participants, including simulation in general, when to become directive, conversational safety, institutional regulations regarding the termination process, working with residents who present personality disorders, inconsistency in feedback to residents, and adapting to the conversational needs of the resident without compromising the integrity of the discussion.

Among EVMS Program Directors and Associates, 23 of 31 (74%) completed the survey to help us prepare the workshop. Respondents represented 19 specialties and had from 1 to 16 years in their current positions. Collaboration was the preferred conflict management style among 91% of the respondents; another 10% rated collaboration as their second preferred choice. In terms of obstacles to managing challenging conversations, 48% of respondents cited passive-aggressive conversational styles, followed by physical aggression (38%), passive/crying (14%), and verbal aggression (4%). Respondents were also asked to rank their comfort level with talking to residents about remediation, probation, and dismissal from a program: 65% ranked termination as least comfortable, 9% rated probation as least comfortable, and 4% rated remediation as least comfortable-in fact, remediation was consistently rated as the least uncomfortable from all respondents. When asked to report challenges when talking with residents about remediation, probation, or termination from a program, 40% of respondents said lack of documentation was their biggest challenge, 30% cited lack of experience/skill, 20% reported threat of litigation, and 10% reported emotional outbursts.

After the workshop, the postencounter survey was sent to all registrants (N=170), and 31 (18%) responded. In terms of their postworkshop increase in awareness of communication style with residents 23% reported that their awareness increased significantly, 36% reported their awareness increased moderately, 20% reported their awareness increased slightly, and 20% reported their awareness stayed the same. The workshop was rated as extremely helpful by 37%, quite helpful by 43%, moderately helpful by 17%, slightly helpful by 3%, and not helpful by 0%. When asked about the usefulness of the standardized resident simulation, 47% rated it as extremely useful, 40% rated is as quite useful, 7% rated it as moderately useful, 7% rated it as slightly useful, and 0% said it was not useful at all. Regarding the amount of simulation included in the workshop, 24% wanted future workshops to include more simulation, 72% wanted the same amount of simulation, and 3% wanted less simulation.

After the workshop, 20% of survey respondents reported that they lacked experience/skills to manage stressful conversations, 30% were concerned by lack of documentation, 23% feared litigation, and 10% were concerned about emotional outbursts; 14% selected Other because they felt equally challenged by multiple selections, particularly their lack of experience/skills. Still other challenges involved residents with professionalism and personality issues.

ACGME sent an 8-item postworkshop survey to 170 participants, 80 (47%) of whom responded. Responses regarding workshop content used a 5-point Likert scale in which 5=very high and 1=very low. Items rated the extent to which the workshop objectives were relevant to participants’ needs, whether the workshop met overall objectives, how the presentation contributed, how the session affected competence and performance, and how the session and speakers rated overall. Mean scores for all questions ranged from 4.44 to 4.61.

## Preparing future workshops

Future work includes development of workshops for program directors and faculty regarding challenging conversations, including smaller workshops to facilitate individual practice and group reflection on challenging conversations. The initial workshop successfully focused awareness on stressful conversations and generated stimulating conversations. We are taking several steps to refine and improve the workshop:

• A future iteration of this workshop is planned for the 2018 ACGME educational conference in Florida and will include more time and simulators.
• At EVMS, the fall program directors’ retreat included a 3-hour version of this workshop as part of general training.
• Further customization may include individual coaching for faculty to emphasize specific program context within specialties.
• Our GME staff offer leadership coaching to residents and faculty on professional communication, conflict management styles, and effective self-care; simulation may be utilized in these coaching sessions.
• Our office of GME will be facilitating a faculty and resident workshop with one of the surgery subspecialties on conflict style and managing difficult conversations.

We welcome expressions of interest and collaboration.

## Take Home Messages

• Discussions with residents regarding remediation, probation, or dismissal are challenging but can be effectively managed.
• Results of the survey showed that the most challenging personality types during stressful conversations were the crying/sad resident, the passive-aggressive resident and the angry/argumentative resident.
• The authors devised a workshop that includes lectures, videos that demonstrate challenging conversations, and group discussions.
• Postworkshop surveys reflected participants’ improved confidence in conducting stressful conversations.
• The authors welcome collaboration as they continue to develop and refine the workshop.

## Notes On Contributors

Agatha Parks-Savage, EdD, RN, LPC, is Assistant Dean, Office of Graduate Medical Education and Associate Professor, Department of Family and Community Medicine, Eastern Virginia Medical School, Norfolk, VA.

Amelia Wallace, MS, MMHPE, is Senior Standardized Patient Educator, Sentara Center for Simulation and Immersive Learning, Eastern Virginia Medical School.

Cynthia P. Cadieux, PhD, RDN, FAND, is Assistant Dean, Institutional Effectiveness and Program Review, Director, Master’s in Medical and Health Professions Education Program, and Director, Distance Education, School of Health Professions, Eastern Virginia Medical School

Linda R. Archer, PhD, is ACGME Designated Institutional Officer (DIO), Vice-Dean, Office of Graduate Medical Education, and Professor, Department of Family and Community Medicine, Eastern Virginia Medical School.
