# Supplementary material for: Managing emotionally charged conversations between faculty and residents: a skill-building workshop
Source: MedEdPublish (2016). 2018 Apr 10;7:80. [Version 1] doi: 10.15694/mep.2018.0000080.1 (PMC10711964; doi:10.15694/mep.2018.0000080.1)
Supplement: Peer review history

# Reviewer report: Reviewer response for version 1

**DOI:** 10.21956/mep.19554.r28983

**Published:** 2019-07-22

**Authors:** Trevor Gibbs

**Affiliations:** 1 AMEE

This review has been migrated. The reviewer awarded 4 stars out of 5 A very interesting paper to read that clearly describes an approach to the development of a much needed skills development workshop. I learned much from this paper, especially how to structure and the content used. Clearly the authors have done a lot of work in this area and researched the topic as well as possible. Although I feel that this is early days and there is the need for more follow-up and extension, I would recommend this for anyone involved either in the area of conflict resolution within Universities / Schools / Institutions and all those involved in faculty development.

Reviewer Expertise:

NA

No decision status is available

# Reviewer report: Reviewer response for version 1

**DOI:** 10.21956/mep.19554.r28981

**Published:** 2019-03-02

**Authors:** Ken Masters

**Affiliations:** 1 Sultan Qaboos University

This review has been migrated. The reviewer awarded 3 stars out of 5 An interesting paper on a very difficult topic of remediation, probation, and dismissal of residents. The authors’ literature review highlights the most important complexities involved. The three cases are described in detail, and, indeed, would represent challenges for staff. It is a pity that the post-encounter survey response was so low. In future, the organisers may consider using a short survey form that can be taken electronically, and taken during the workshop on participants’ mobile devices.In addition, although a more thorough evaluation of the workshop would require more detailed follow up with the participants, the authors have made it clear that this was a pilot. As a pilot, it can be deemed to be successful, and I would be interested to read about later developments and instances of this workshop.

Reviewer Expertise:

NA

No decision status is available

# Reviewer report: Reviewer response for version 1

**DOI:** 10.21956/mep.19554.r28982

**Published:** 2018-04-11

**Authors:** Omayma Hamed

**Affiliations:** 1 King Abdulaziz University

This review has been migrated. The reviewer awarded 2 stars out of 5 The study addresses a clearly focused issue. The research method was anchored in theoretical frameworks and which guided the authors to build their survey upon which the workshop was designed. Although the response rate for the first survey upon which the workshop was designed was satisfactory (74%), yet it was very low concerning the post-encounter survey (18%) and the post- workshop evaluation survey (47%). In the latter two cases, non-respondents bias could affect the validity of results. In addition, evaluation of the workshop did not follow an evaluation model. It only measured tier-1 of Kirkpatrick’s evaluation model. Impact of the workshop on the target participants could not be evaluated by only using a post-encounter survey without comparing it to a pre-encounter one; and a post-workshop survey is not enough to evaluate the effectiveness of the workshop.

Reviewer Expertise:

NA

No decision status is available
